# Therapeutic Targeting of the NRF2 Signaling Pathway in Cancer

**DOI:** 10.3390/molecules26051417

**Published:** 2021-03-05

**Authors:** Pelin Telkoparan-Akillilar, Emiliano Panieri, Dilek Cevik, Sibel Suzen, Luciano Saso

**Affiliations:** 1 Department of Medical Biology, Faculty of Medicine, Yuksek Ihtisas University, 06520 Ankara, Turkey; pelintelkoparan@gmail.com (P.T.-A.); cevikdi@gmail.com (D.C.); 2 Department of Physiology and Pharmacology, Faculty of Pharmacy and Medicine, “Vittorio Erspamer”, Sapienza University of Rome, 00185 Rome, Italy; emiliano.panieri@hotmail.it; 3 Department of Pharmaceutical Chemistry, Faculty of Pharmacy, Ankara University, 06560 Ankara, Turkey; sibel.suzen@pharmacy.ankara.edu.tr

**Keywords:** Nrf2, Keap1, cancer, oxidative stress

## Abstract

Cancer is one of the most fatal diseases with an increasing incidence and mortality all over the world. Thus, there is an urgent need for novel therapies targeting major cancer-related pathways. Nuclear factor-erythroid 2-related factor 2 (NRF2) and its major negative modulator Kelch-like ECH-associated protein 1 (KEAP1) are main players of the cellular defense mechanisms against internal and external cell stressors. However, NRF2/KEAP1 signaling pathway is dysregulated in various cancers, thus promoting tumor cell survival and metastasis. In the present review, we discuss the mechanisms of normal and deregulated NRF2 signaling pathway focusing on its cancer-related functions. We further explore activators and inhibitors of this pathway as cancer targeting drug candidates in order to provide an extensive background on the subject.

## 1. Introduction

Cancer is a non-communicable disease with an increasing incidence and mortality in many countries worldwide, which is expected to become the leading cause of death in every continent by the end of this century. According to the global statistical data published by (World Health Organization (WHO), Geneva, Switzerland) the main reason behind the growth of cancer-related deaths is the expansion of the world’s aging population [1]. Another critical phenomenon is that common cancer profiles have been changing in a way that infection and or poverty-related cancers tend to decrease while cancers that are associated with Westernized lifestyle tend to augment [2]. In this context, it is of great importance that researchers define the roles of critical molecular players related to tumor formation, progression, and metastasis. At the molecular level, cell division and death of damaged/mutated cells are tightly controlled by several pathways in order to prevent survival of damaged cells bearing mutations and pass those mutations to the next generations. Sometimes a critically damaged cell can take a life-changing decision and takes steps to secure its own survival despite the cost of transforming into a tumor cell. The steps to be taken in the way of transformation by a precancerous cell are described in the highly cited Weinberg review in detail [3]. Genes and signal transduction pathways common to more than one hallmark of cancer recently gained extra attention as promising therapeutic targets. Among the others, redox signaling emerged as a signal transduction pathway involved in every step of carcinogenesis [4]. Nuclear factor erythroid 2-related factor 2 (NRF2), also known as NFE2L2, is considered as the leading transcription factor controlling cellular redox homeostasis and antioxidant pathways [5]. Being the major stress regulator of the cell, NRF2 is involved in tumor formation, progression, and metastasis [6]. For this reason, a large number of studies is currently ongoing to better characterize NRF2 pathway and its roles in cancer. NRF2 displays a complex behavior in carcinogenesis [7]. To fully address the importance of NRF2 pathway in cancer, it is necessary to provide a description of its negative regulator KEAP1, that interacts with NRF2 to downmodulate its expression in cells and strictly control cellular homeostasis [8]. Under normal or low/moderate stress conditions, there is a tight balance between KEAP1 activity and NRF2 protein levels, which provides regulated antioxidant response, detoxification, and prevention of cancer. However, excessive stress, continuous overexpression of NRF2 or downregulation of KEAP1 cause a shift in this balance, which, in turn, acts in favor of carcinogenesis. Unraveling these roles would provide researchers to target NRF2 pathway in a more selective way to fully eradicate cancer without promoting its pro-oncogenic functions also known as the “dark side” of NRF2 [9]. In this respect, experimental studies wherein NRF2 function was abrogated with genetic or chemical approaches, support the notion that this transcription factor plays a cytoprotective role, acting as a tumor suppressor in specific contexts [10,11]. It has also been reported that NRF2 loss is strongly associated with tumor malignancy and metastatic behavior of cancer cells [12]. Moreover, partial or complete depletion of KEAP1 has been shown to promote cancer initiation and growth suggesting that KEAP1 can be also regarded as a tumor suppressor, similarly to NRF2 [13,14]. Based on collective data on NRF2 and KEAP1, there is a growing interest in better defining the therapeutic use of natural obtained or chemically synthesized activators of NRF2 with tumor-suppressing properties [15]. Yet, none of these compounds, either from natural or chemical sources, showed a consistent, stable, and dose-dependent effect to become a solid anticancer drug candidate, due to the context-dependent effects of NRF2 pathway in tumors [16]. Moreover, many studies reported that the abnormal activation of NRF2 is a common event in tumor cells, caused by several factors like somatic mutations, oncogenic signaling, epigenetic changes, metabolic reprograming and altered redox balance in cancer cells [17]. Indeed, abnormal NRF2 expression has been detected in various tumors such as lung, esophageal, laryngeal, skin, pancreas and liver cancers [18]. Despite these observations might argue against the assumption of NRF2 being a tumor suppressor, they actually indicate that NRF2, is a context-dependent transcription factor that can act as an oncogene under certain circumstances. In addition, abnormal KEAP1 expression has been observed in several cancers including lung, liver, pancreas, and ovarian cancers [19]. Thus, based on these data, it appears that there is a fine-tuning between KEAP1 and NRF2 levels and this determines which effect of this pathway will be more prominent under specific circumstances of a certain type of tumor. On the other hand, extensive research is focusing on NRF2 inhibitors in consideration of its cancer promoting roles, especially in the later stages of tumorigenesis [16]. However, these inhibitors may lack specificity and therefore interact with other downstream pathways causing undesired effects. Thus, it is of great importance that these drug candidates will be meticulously tested in large clinical studies in order to identify the specific cohorts of patients and the clinical context most likely having beneficial effects and minimal side toxicity. To sum up, in the upcoming sections of this review, we will describe more in detail the structural elements of the NRF2-KEAP1 pathway, the mechanisms underlying the tumor-suppressive and oncogenic properties of NRF2 and KEAP1 proteins as well as both activators and inhibitors of the NRF2 pathway.

## 2. NRF2 and KEAP1 Signaling Pathway

**Figure 1:**
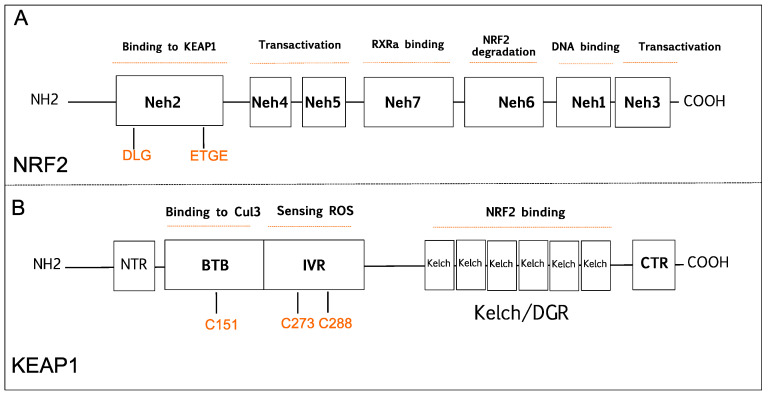
Schematic diagram is showing domain structures of nuclear factor E2-related factor 2 (NRF2), and Kelch-like ECH-associated protein 1 (KEAP1). (**A**) NRF2 has seven domains (Neh1-7). The Neh1 domain is responsible for DNA binding. The Neh2 domain contains DLG and ETGE motifs that are critical for KEAP1 binding. The Neh3, Neh4, and Neh5 are known transactivation domains. The Neh6 is important for proteasomal degradation of NRF2. The Neh7 domain is responsible for RXRα binding. (**B**) KEAP1 has five domains; NTR, CTR, BTB, Bric-a-Brac domain binds to Cul3 that is critical for KEAP1 dimerization; IVR, intervening region contains cysteine residues 273 (C273) and 288 (C288) that are important for sensing reactive oxygen species (ROS); the Kelch/DGR domain is important for NRF2 binding. BTB, Broad complex/Tramtrack/Bric-a-Brac; CTR, carboxy-terminal domain; Cul3, Cullin E3 ubiquitin ligase; IVR, Intervening region; Neh, NRF2-ECH homology; NTR, N-terminal region; RXRα, retinoid X receptor-alpha.

NRF2 was discovered in 1994 and belongs to the Cap and Collar (CNC) basic-region leucine zipper transcription factor family [20]. NRF2 has seven conserved NRF2-ECH homology domains comprising Neh1 to Neh7 (Figure 1A). Neh1, Neh3, Neh4, and Neh5 domains are involved in the transcriptional activation of NRF2 by binding its co-activators. Neh2, Neh6, and Neh7 control the stability of NRF2 through responding as a negative regulatory domain [16]. Neh1 domain is known as a CNC-bZIP domain that allows NRF2 to bind antioxidant response element (ARE), also known as the electrophile response element (EpRE) through interaction with other factors like small musculoaponeurotic fibrosarcoma (sMAF) [21]. Neh2 domain functions as a major regulatory domain of NRF2 containing ETGE and DLG regions that are required for the interaction with KEAP1. In addition, Neh2 domain has lysine rich residues responsible for the ubiquitination and subsequent proteasomal degradation of NRF2 [22]. Neh3 is the transactivation domain recruiting co-activators that are necessary for the transactivation of NRF2 [23]. NRF2 also possesses Neh4 and Neh5 domains containing acid-rich residues that interact with CREB-binding protein with histone acetyltransferase activity (CBP) [24]. The Neh6 domain contains serine-rich residues that can be phosphorylated by Glycogen Synthase Kinase 3b (GSK-3β) and leads to proteasomal degradation of NRF2 through cullin 1 (Cul1)-dependent ubiquitination [25]. The Neh7 domain mediates the binding of RXRα (retinoid X receptor α) that inhibits the NRF2 transcriptional activity [26].

KEAP1 was identified as a negative regulator of NRF2 that consists of five functional domains, namely, the N-terminal domain, Broad complex/Tramtrack, Bric-a-Brac domain (BTB), a cysteine-rich intervening region (IVR), Kelch domain, or double glycine repeat (DGR), and carboxyterminal domain (Figure 1B) [27]. The BTB domain is required for homo dimerization of KEAP1 and plays a critical role in ubiquitination of NRF2 through the interaction with the CUL3-based E3 ubiquitin ligase complex [28]. The IVR domain has highly reactive cysteine residues that are responsible for sensing reactive oxygen species (ROS), reactive nitrogen species (RNS), and hydrogen sulfide (H_2_S) [29,30,31]. The Kelch/DGR domain functions as an NRF2 repressor, and it contains of six Kelch motif repeats, which are required for interaction with Neh2 domain of NRF2 [32].

NRF2 increases cellular antioxidant capacity by controlling the expression of detoxifying and antioxidant genes. Hence, NRF2 has been previously known as a transcription factor that inhibits cancer development. Under homeostatic conditions, KEAP1 plays a critical role in NRF2 activity by binding to DLG/ETGE motifs in the Neh2 domain, and keeps NRF2 protein at low levels in the cytoplasm by promoting its polyubiquitylation and proteasomal degradation [33]. However, under stress conditions, highly reactive cysteine residues in KEAP1 are oxidized, and this modification disrupts the binding of KEAP1 to NRF2 promoting its nuclear translocation, wherein NRF2 forms NRF2-sMaf heterodimers via its Neh1 domain and induces gene expression by binding to the ARE sequences in the promoter regions of NRF2 target genes [33].

KEAP1-NRF2 pathway is one of the major signaling cascades that promote antioxidant defense in normal cells, which is a crucial mechanism in the prevention of cancer development. Many studies have shown that KEAP1 and NRF2 proteins function as tumor suppressors, as their absence leads to tumorigenesis while other work indicates that NRF2 can also promote tumor progression. In the following sections, we will briefly discuss past and present studies focused on this seemingly paradoxical aspect.

### 2.1. The Tumor Suppressive Role of NRF2 Pathway

A strong indication supporting a tumor-suppressive role of the NRF2 signaling derives from a number of in vivo studies comparing the sensitivity to chemically induced carcinogenesis in NRF2-knockout mice (Nfe2l2-/-) and wild-type mice. In this respect, it was found that NRF2-null mice are more susceptible to developing bladder, skin, and stomach cancer when exposed to chemical carcinogens compared to wild-type mice [34]. In addition, the basal expression level of ARE-mediated genes such as GCL, GST, HMOX1, NQO1, and UGT was found to be significantly suppressed in NRF2-deficient mice compared to the wild-type counterpart [11,35,36]. The mechanism, by which NRF2 protects cells from chemical-induced carcinogenesis, appears to depend on its role in detoxification of chemical carcinogens and ROS, and the induction of DNA damage repair mechanisms that ultimately prevent mutations. In a study with mice harboring SNPs (single nucleotide polymorphisms) in the promoter region of the NRF2 gene, it was shown that reduced NRF2 expression made mice more susceptible to lung injury due to hypoxia [37]. SNP-bearing individuals have lower NRF2 mRNA levels causing an elevated risk of developing non-small cell lung cancer (NSCLC) [13,14].

### 2.2. The Tumor Suppressive Role of KEAP1

To investigate the direct role of KEAP1 in tumor development, Wakabayashi et al. generated Keap1-knockout mice (Keap1-/-) [38]. Unfortunately, these animals developed hyperkeratosis of the esophagus and fore stomach and showed postnatal lethality within the first 3 weeks [38]. Moreover, Keap1-deficient mice demonstrated upregulation of detoxifying enzymes, including GST and NQO1, and higher NRF2 signaling before death [38]. KEAP1 was also reported to target NRF2/S100P (S100 calcium-binding protein P) pathway in non-small cell lung cancer (NSCLC) cells, acting as a tumor suppressor. Thus, it was suggested that KEAP1 can be used as a biomarker to monitor NSCLC progression [39].

### 2.3. The Carcinogenic Role of NRF2 

**Figure 2:**
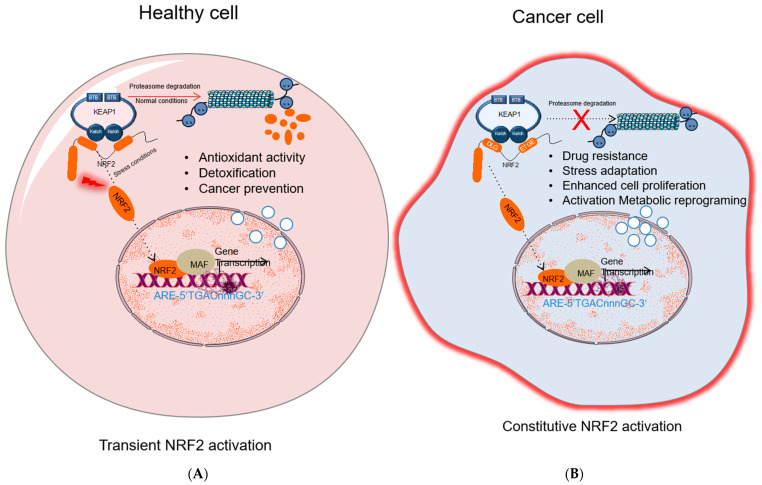
NRF2 activation in healthy and cancer cells. (**A**) In healthy cell, under normal conditions NRF2 level is inhibited by KEAP1-mediated proteasomal degradation; under stress conditions, NRF2 dissociates from KEAP1, accumulates in nucleus and activates cytoprotective gene expression. (**B**) In cancer cells, different molecular mechanisms cause constitutive NRF2 activation that results in drug resistance, stress adaptation, cells proliferation, and activation of metabolic reprogramming and induces expression of genes related to tumor progression. NRF2, nuclear factor erythroid 2-related factor 2; KEAP1, Kelch-like ECH-associated protein 1; MAF, small musculoaponeurotic fibrosarcoma protein; Pol II; RNA polymerase II; ARE: antioxidant response element.

While many studies show that activation of NRF2 protects normal cells against various toxic substances and diseases, it has been shown that the overactivation of NRF2 also supports cancer progression and protects cancer cells from oxidative damage leading to chemoresistance and radioresistance (Figure 2). Elevated levels of NRF2 in cancer induce the upregulation of glucose 6-phosphate dehydrogenase (G6PD), transketolase (TKT), 6-phosphogluconate dehydrogenase (PGD), and other metabolic enzymes [40]. The augmented activation of these metabolic enzymes increases the synthesis of purine and amino acids and refills the NADPH pool via the pentose phosphate pathway (PPP) leading to metabolic reprogramming for cell proliferation and enhanced antioxidant capacity. Moreover, NRF2 regulates the basal expression of Mdm2, a direct inhibitor of p53 [41]. Therefore, increased Nrf2 expression indirectly downregulates p53 and contributes to tumor survival by suppressing p53-related apoptotic signals.

Recent studies have demonstrated that NRF2 plays a critical role in promoting intrinsic and acquired chemoresistance of cancer cells to common chemotherapeutics by activating drug resistance proteins and drug transporters such as UDP-glucuronosyl-transferase 1A1 (UGT1A) and multidrug-resistance-associated protein-1 (MRP1). In a study conducted in human doxorubicin-resistant ovarian cancer cells, NRF2 level was found to be elevated compared to the control cell line, and silencing of NRF2 expression via siRNA restored drug sensitivity [42]. In another study, chemical activation of NRF2 provided a survival advantage to neuroblastoma cells in response to cancer drugs such as cisplatin, doxorubicin, and etoposide [43]. Based on these findings, Cho et al. demonstrated that depletion of NRF2 expression via siRNA knockdown increased the effectiveness of cisplatin in ovarian cancer cells [14].

Moreover, persistent activation of NRF2 was reported to attenuate the toxicity of ionizing radiation and drug treatment in human lung cancer cells, while NRF2 knockdown enhanced cellular response to ionizing radiation and chemotherapeutic drugs. These findings suggest that targeting NRF2 activity alone or in combination with other drugs could be an effective strategy to improve the sensitivity of malignant cells to anticancer therapies [44,45].

Furthermore, recent studies demonstrate the role of NRF2 in tumor metastasis. Constitutively active NRF2 promotes lung cancer via inhibiting degradation of a pro-metastatic transcription factor Bach1 [46]. Overexpression of NRF2 in breast cancer leads to cell proliferation and metastasis by activating the RhoA gene and its downstream signal proteins [47]. The critical role of NRF2 in tumor metastasis and proliferation has been shown in human hepatocellular carcinoma via regulating expression of Bcl-xL and Metalloproteinase-9 (MMP-9) genes [48]. NRF2 also activates epithelial mesenchymal transition (EMT) and invasion in pancreatic adenosquamous carcinoma cells by decreasing E-cadherin gene expression [49]. In addition, depletion of NRF2 decreases radiation-induced NSCLC invasion through promoting E-cadherin expression and reducing N-cadherin and MMP2/9 expression [50].

### 2.4. The Carcinogenic Role of KEAP1

Despite protective effects on cancer progression, studies have also demonstrated the carcinogenic role of KEAP1 mutations in various cancers such as gallbladder, prostate, liver, colorectal, lung, breast, and prostate cancers [51,52,53,54]. Some mutations found in the N-terminal and BTB domains of KEAP1 prevented ubiquitination of NRF2 through the disruption of KEAP1-CUL3 formation, and other mutations in the Kelch domains inhibited interaction of KEAP1-NRF2 and caused stabilization of NRF2 [45,46,55]. Additionally, mutations in KEAP1 were also detected in liver and gallbladder, which caused over expression of antioxidant and phase II detoxification enzymes that have roles in cancer chemo-resistance [53,56].

## 3. NRF2 Activation Mechanisms in Cancer

**Figure 3:**
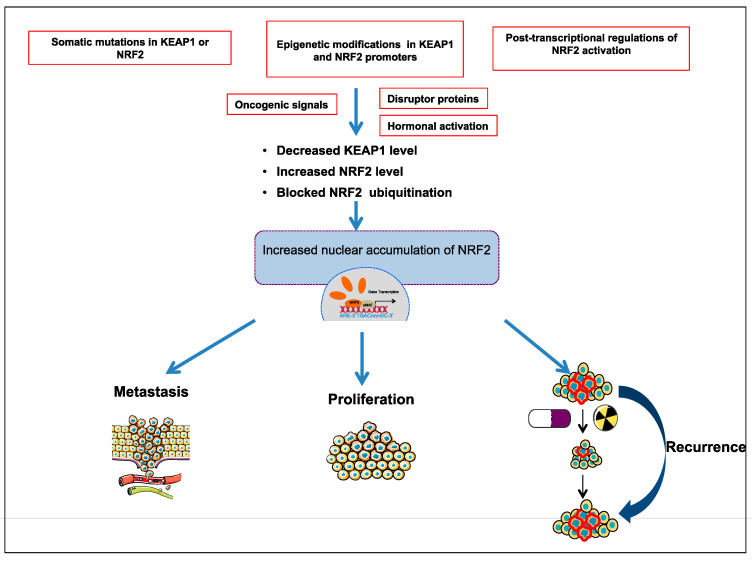
Different molecular mechanisms are responsible for activation of NRF2-KEAP1 pathway in cancer; somatic mutations in KEAP1 or NRF2; epigenetic modifications in KEAP1 and NRF2 promoter; post-transcriptional activation of NRF2; oncogenic signals; hormonal activation.

Comprehensive studies validated that NRF2-KEAP1 signaling pathway is activated in several cancers such as skin, lung, bladder, hepatocellular carcinoma, esophagus, ovarian, prostate, pancreatic, and breast cancer [6,52,53,55,57]. The molecular mechanisms responsible for the activation of NRF2 in cancer are schematized in Figure 3 and further details are discussed below:

### 3.1. The Somatic Mutations in KEAP1 or NRF2

In cancer, somatic loss of function mutations in *KEAP1* or *NFE2L2* genes are the most known mechanisms that reduce NRF2-KEAP1 binding and prevent degradation of NRF2 through KEAP1/CUL3/RBX1 E3-ubiquitin ligase complex [45,51]. Increasing evidence has established that the inhibition of NRF2-KEAP1 interaction leads to the overexpression of NRF2 in cancer cells that, in turn, enhances the activation of antioxidant defense system, and proteins involved in chemoresistance and radioresistance system via activating ARE-containing gene expression. Most of the inactivating mutations in the *NFE2L2* gene were detected within ETGE and DLG motifs in various cancers such as lung, head, neck, and esophageal carcinoma [53]. It was also reported that the exon2 loss of the *NF2EL2* pre-mRNA abolishes the KEAP1–NRF2 protein–protein interaction, thereby inducing NRF2 accumulation and transcriptional activation of its target genes in lung, head, and neck cancers [58].

Inactivating mutations in *KEAP1* gene occur frequently in many cancer types and largely affect the NRF2-KEAP1 interaction. Unlike *NFE2L2*, *KEAP1* mutations can be missense or nonsense mutations and observed on the entire gene [45,55]. Some of the mutations in *KEAP1* gene lead to deregulation of apoptosis, autophagy, and inflammation by accumulation of BCL2 and p62 proteins [59,60]. The first loss-of-function mutations in Kelch/DGR domain of KEAP1 were reported in human lung adenocarcinoma cell lines [54]. Then, somatic mutations in Kelch/IVR domain of KEAP1 were detected in both human NSCLC cell lines and clinical NSCLC patients’ tumor samples [45,55]. Recently, different research groups also reported that *KEAP1* genetic alterations could be novel molecular hallmarks in high neuroendocrine gene expressing lung cancers [61,62].

### 3.2. Epigenetic Modifications in KEAP1 and NRF2 Promoters

Besides somatic mutations, epigenetic changes at *KEAP1* and *NFE2L2* promoters may promote to the accumulation of NRF2 and depletion of KEAP1 in cancer cells. Several studies indicate that epigenetic mechanisms play a role in the regulation of KEAP1/NRF2 signaling. In particular, silencing of *KEAP1* by different epigenetic mechanisms in many tumors causes NRF2 accumulation. In lung, colon, and prostate cancers, *KEAP1* promoter was found to be significantly hypermethylated [43,63,64,65]. Moreover, hypermethylation within the promoter region of *KEAP1* was associated with poor clinical prognoses in patients with glioma [66]. On the other hand, it has been shown that *NFE2L2* promoter demethylation resulted in NRF2 accumulation and chemoresistance in colon cancer cells [67]. Therefore, from a therapeutic perspective, *KEAP1* methylation or *NFE2L2* demethylation can be targeted to inhibit abnormal NRF2 expression in different cancers.

### 3.3. Post-Transcriptional Regulation of NRF2 Activation

MicroRNAs (miRNAs) are small, 19–25 nucleotides in length, non-coding RNA molecules that play roles in regulating gene expression by sequence-specific binding to mRNA sequences [68]. Several studies concluded that KEAP1 and NRF2 levels can be regulated at the post-transcriptional level in different cancers by abnormal expression of miRNAs targeting these genes. For example, miR-507, miR-634, miR-450a, and miR-129-5p directly target and suppress NRF2 activity. Studies have shown that these miRNAs are downregulated in esophageal squamous cell carcinoma (ESCC) and lead to upregulation of NRF2 mRNA [69].

Furthermore, miR-27a, miR-141, miR-144, miR-153, miR-200a, miR-432, and miR-23a modulate *KEAP1* mRNA expression and induce NRF2 activation [70]. It was reported that miR-141 is overexpressed in breast and ovarian cancer, and additionally, overexpression of this miRNA increased chemoresistance of HCC cells to 5-fluorouracil through the activation of NRF2-driven antioxidant pathways [71,72].

### 3.4. Disruptor Proteins

Several disrupting proteins are involved in the activation of NRF2 in cancer. Moreover, p62, also known as sequestosome 1 (SQSTM1), is an autophagy receptor protein that contains the STGE motif, which is similar to the ETGE motif of NRF2. This protein competes with NRF2 for KEAP1 binding and promotes autophagic degradation of KEAP1 [73,74,75,76]. Studies proved that when p62 expression was decreased by siRNA-mediated knockdown, NRF2 and its target genes were downregulated, while the half-life of KEAP1 increased by twofold [73,76]. In addition, elevated p62 contributed to renal cancer progression and hepatocellular carcinoma through the activation of NRF2 [77,78,79]. These studies emphasize the critical role of p62 and NRF2 axis in the regulation of tumor development. 

Besides, p21, which is a direct target of p53, associates with ETGE and/or DLG motifs in NRF2 and disrupts NRF2-KEAP1 binding causing NRF2 accumulation [80]. Furthermore, Wilms tumor gene on the X chromosome (WTX) and partner and localizer of BRCA2, also known as PALB2 proteins have been shown to bind KEAP1 and suppress NRF2 ubiquitination [81,82]. Similarly, the protein dipeptidyl peptidase 3 (DPP3) was shown to inhibit NRF2 ubiquitination through binding to KEAP1, thus activating NRF2-dependent gene transcription in breast cancer [83].

### 3.5. Oncogenic Signals

Oncogenic signals contribute to abnormal NRF2 activation in cancer through transcriptional upregulation of NRF2. DeNicola et al. reported that NRF2 level can be increased by the activation of oncogenic alleles of *KRAS*, *BRAF*, and *C-MYC* (KRASG12D, BRAFV619E, and C-MYCERT12) [84]. In addition, they also demonstrated that K-RAS and B-RAF activated Jun and Myc transcription factors, which, in turn, promoted cancer cell survival and chemoresistance [84]. Similarly, disruption of tumor suppressor phosphatase and tensin homologue (PTEN) protein-activated NRF2 in human cancers [85]. Moreover, KRAS-ERK signaling pathway plays a critical role in elevation of NRF2 transcription via 12-O Tetradecanoylphorbol-13-acetate (TPA) response element that localizes in a regulator site in NRF2 exon1 [86]. Furthermore, the phosphatidylinositol-4,5-bisphosphate 3-Kinase (PI3K) serine/threonine kinase (AKT) signaling pathway also upregulated NRF2 transcription through the inhibition of GSK3-β-TrCP-induced proteasomal degradation of NRF2 [87]. 

### 3.6. Hormonal Activation

Several studies validated the effects of hormonal activation of NRF2 on cancer progression. Gonadotropins and sex steroid hormones, including follicle-stimulating hormone (FSH), estrogen (E2), and luteinizing hormone (LH), have been reported to be critical in activation of NRF2 through the induction of ROS that inhibit KEAP1 by oxidation of its cysteine residues [88]. In addition, follicle-stimulating hormone (FSH) is known to induce expression of vascular endothelial growth factor (VEGF) and hypoxia inducible factor 1α (HIF1α). Thus, FSH contributes to tumor angiogenesis through ROS-mediated NRF2 signaling [89].

## 4. NRF2 Modulators for Cancer Therapy 

### 4.1. NRF2 Activators 

Mostly, activation of NRF2 has been observed as therapeutic, but current research has revealed that activation process can be pro-oncogenic as well, depending on the status of NRF2 activation. A recent study indicates, that oncogenic signaling may affect the behavior of NRF2 by increasing its mRNA levels. Depending on this, the oncogenic activation of K-RAS and B-RAF are sufficient to increase the NRF2 mRNA levels and support ROS detoxification in human cancer cells [84]. Targeting NRF2 might represent an effective therapeutic approach in solid tumors with aberrant activation of the K-RAS signaling related to chemoresistance [15]. Under normal physiological conditions, NRF2 interacts with two molecules of KEAP1 by its Neh2 ETGE and DLG motifs to trigger Cullin 3 (Cul3)-based E3 ligase complex-mediated NRF2 ubiquitination reaction [90]. By the effect of oxidative stress or anti-cancer compounds, the cysteine residues of KEAP1 are chemically modified leading to degradation of NRF2. The binding capacity between NRF2 and KEAP1 is reduced. This allows the de novo-synthesized NRF2 to enter into nucleus and interact with the antioxidant element (ARE) in multiple genes [91]. 

Presently, recognized NRF2 activators such as curcumin, sulforaphane, and oltipraz have been found not target specific and might increase risk of “off-target” toxicity due to their capability to interact with the cysteines of other enzymes and proteins. Likewise, molecular instability, lower membrane permeability, and poor bioavailability of some NRF2 modulators are another important reason of concern. Demanding the modulators of NRF2-KEAP1 pathway, therefore, requires not only potent efficacy but also good bioavailability and specificity [7].

**Figure 4:**
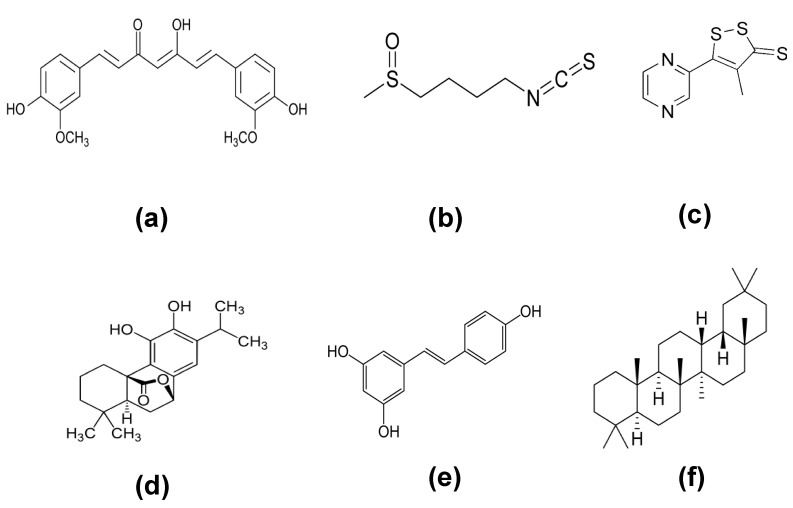
Some NRF2 activator molecules. (**a**) Curcumin, (**b**) sulforaphane, (**c**) oltipraz, (**d**) carnosol, (**e**) resveratrol, and (**f**) oleanane.

Some molecules from natural sources like carnosol and resveratrol and synthetically developed like oltipraz and oleanane triterpenoids (Figure 4) have been found to exert a chemopreventive role by inducing NRF2/ARE-regulated genes. Their mechanism of action usually affects NRF2 activity by changing intermolecular disulfide bonds between two KEAP1 molecules at Cys273 and Cys288, to increase NRF2 nuclear accumulation [11].

Qin et al. published in a systematic review that many of the NRF2 activators, such as MT477 [92], oleanolic acid (OA), fisetin, QD325 [93], resveratrol, sulforaphane (SFN), and alpha lipoic acid have been intending to increase NRF2 expression and/or enhance NRF2 action [94]. OA and SFN have been proved to stimulate the upstream regulators of the NRF2 pathway, ERK, and AMPK (AMP-activated protein kinase), respectively, which play a causal role in the increased expression of NRF2 [95,96].

The abnormal expression of KEAP1 or NRF2 has been often observed in many cancer types. Despite strong evidence indicate that NRF2 could prevent cells from oncogenesis as a tumor suppressor, researchers found that it might promote cancer progression and resistance to chemotherapeutic drugs as an oncogene as well. Tong et al. found that the activation of NRF2 signaling by some chemicals was able to block tumor development or decrease chemoresistance [97]. Tsai et al. proved that miconazole markedly enhanced NRF2 activation in bladder cancer cells [98]. Miconazole-induced production of ROS stimulated NRF2 activation. A study revealed that the promoter of *Il17d*, containing an ARE, is controlled by NRF2 and that physiological stimulation of NRF2 could thus encourage tumor rejection by employment of natural killer cells [99]. Generally, these findings exposed that NRF2 expression is observed in cancer development via preservation of immune system, whereas NRF2 in cancer cells supported tumor growth.

It was shown that NRF2 might stop DNA damage secondarily by decreasing the levels of ROS [100]. Tao et al. recognized that NRF2 activation protects from UV damage by reducing ROS [101]. 

Considering the occurrence and the role of NRF2 in cancer, it has been proposed that targeting this transcription factor might be a significant therapeutic approach. NRF2 activators could be effective to prevent chemical carcinogenesis, while NRF2 inhibitors might be used in cancer treatment [6]. 

The present consent is that NRF2 activators stabilize NRF2 and promote its translocation by preventing KEAP1 binding to NRF2 [102]. The possible pharmacological action of NRF2 activators in prevention and treatment of cancer in which oxidative stress plays a causal role in the pathogenesis is still under investigation.

### 4.2. NRF2 Inhibitors

As already mentioned, NRF2 can exert a dualistic role in cancer acting as an oncogene or an oncosuppressor requiring a careful consideration of the specific context of its activation to fully exploit its clinical potential [9]. There is, however, unanimous consent that the cytoprotective program controlled by NRF2 might confer selective advantages to cancer cells promoting their adaptation to adverse growth conditions. Indeed, a large body of evidence supports the notion that NRF2 overactivation is strictly associated with tumor invasiveness, distant metastasis formation, therapy resistance, and poor clinical outcomes in many cancer patients [6]. Based on this, the identification of NRF2 inhibitors is rapidly emerging as a promising anticancer strategy in solid and hematologic cancers characterized by persistent NRF2 activation. It has to be recognized that specific and selective NRF2 inhibitors are still lacking, so that great efforts have been made to find alternative and effective strategies to impair NRF2 signaling acting at different levels of its biological regulation [103]. For instance, agents interfering with the transcription of the NFE2L2 gene such as themozolomide-1 or homoharringtonine have been successfully employed to promote chemosensitivity in different types of tumors through altered redox balance [104,105]. Other compounds such as apigenin, omipalisib, and entinostat have been used to block the NRF2 mRNA translation by direct or indirect mechanisms, promoting anticancer effects in experimental models of hepatic cancer [106], gastric cancer [107], as well as sarcoma and osteosarcoma [108], respectively. Other researchers took advantage of global repressors of protein synthesis such as brusatol or halofuginone to facilitate NRF2 degradation and prevent the refilling of its intracellular content at the post-transcriptional level in solid and hematologic tumors. By doing so, potent tumor suppressive effects and enhanced chemosensitivity largely derived by overwhelming ROS accumulation, were observed in pancreatic cancer cells [109], colorectal cancer cells [110], acute myeloid leukemia (AML) cells [111], and non-small cell lung cancer (NSCLC) cells [112]. Of note, halofuginone bromide is currently under phase-1 evaluation in a clinical trial conducted on patients with advanced solid tumors (NCT00027677). Along similar lines of evidence, another strategy has focused on the use of compounds that affect NRF2 stabilization and/or enhance its degradation. For instance, the PI3K-DNAPK inhibitor PIK-75 was found to promote NRF2 degradation and overcome gemcitabine resistance in pancreatic cancer cells [113], while the Bcl-2 inhibitor venetoclax was shown to suppress the overexpression of NRF2 induced by hypomethylating agents in AML cells, by promoting NRF2 proteasomal degradation through the reinforced interaction with KEAP1, thus triggering mitochondrial ROS generation and apoptosis [114]. In another experimental context, the natural compound convallatoxin was identified as a potent inhibitor of NRF2 function promoting GSK-3β/β-TrCP-dependent NRF2 degradation in NSCLC cells and restoring their responsiveness to 5-fluorouracil [115]. Promising results were also observed with several compounds impairing NRF2 nuclear translocation and subsequent target genes induction. For instance, the alkaloid trigonelline has been shown to abrogate NRF2 nuclear accumulation in pancreatic cancer cells, and restore their sensitivity to anticancer agents both in vitro and in vivo [116]. Similarly, earlier studies conducted on the flavonoid luteolin, revealed that this natural compound was able to impair NRF2-dependent antioxidant genes induction and increase the chemosensitivity of NSCLC cells by suppressing NRF2 nuclear translocation and reducing the intracellular GSH content [117]. Later evidence consistently reported that luteolin could abrogate NRF2 nuclear translocation and induce ROS overproduction leading to the activation of the intrinsic apoptotic pathway in colorectal cancer cells [118]. Importantly, on the basis of the promising pre-clinical results, luteolin efficacy has been evaluated in a phase-1 clinical trial on patients with tongue squamous cell carcinoma (NCT03288298). Lastly, another promising category of NRF2 inhibitors is represented by agents that affect NRF2 binding to the DNA responsive (ARE) sequences of target genes, which is the most downstream event in the cascade of NRF2 activation. In this regard, one interesting example is represented by ML385, a small molecule inhibitor, which was found to suppress NRF2-MAF binding and to chemosensitize NSCLC cells in vitro as well as in NSCLC animal models, in the context of KEAP1 inactivation [119]. Similarly, the vitamin A derivative known as all-trans retinoic acid (ATRA), has been successfully used to enhance the sensitivity of chemoresistant neuroblastoma cells to bortezomib [120] or to increase the cytotoxic effects of arsenic trioxide in promyelocytic as well as acute myeloid leukemia cells [121]. Mechanistically, data from solid and hematologic cancers have shown that ATRA can repress NRF2 transcription or prevent its nuclear translocation by promoting its association with the nuclear receptor RARα [121,122], which appears to be independent on the antagonistic formation of heterodimers with RXRα [122]. The clinical efficacy of ATRA has been already proven in a consistent number of phase 1–4 clinical trials conducted on solid tumors and hematologic tumors, yet the therapeutic impact of ATRA administration in patients cohorts with NRF2 overactivation still waits to be fully elucidated. Taken together, these data demonstrate that inhibition of NRF2 signaling might represent a valid therapeutic option in those malignant tumors wherein genetic, epigenetic, or upstream oncogenic alterations lead to persistent NRF2 overactivation. Despite, the lack of specific and selective NRF2 inhibitors still represents a significant limitation to the use of alternative strategies of NRF2 inhibition, it is clear that the appropriate understanding of the clinical context, wherein these agents are supposed to exert the most beneficial effects, will pave the way to novel and tailored anti-cancer therapies.

## 5. Conclusions/Perspective

It is known that the NRF2/KEAP1 signaling pathway is a crucial actor in modifying oxidative stress, and the association between NRF2 and cancer is certain. Still, the dual role of NRF2 in cancer has to be considered when developing novel NRF2 modulators [123]. Low concentrations of NRF2 action caused augmented intracellular ROS, harmful effects to cellular molecules such as DNA, proteins, and lipids, as well as apoptosis [124]. Dissimilarly, high concentrations of NRF2 caused cellular resistance to ROS and metabolic stress [125]. Therefore, mutations and modifications that escalate NRF2 activity are associated to cancer and chemo- and radioresistance developments [126].

Many studies propose that suppression of NRF2-related antioxidant mechanisms may show a hopeful therapeutic methodology to activate ROS-dependent cell death in numerous cancer types. Developing the modulators of Nrf2-Keap1 pathway, thus, obliges not only strong efficacy but also good bioavailability and specificity.

NRF2 modifies the expression of proteins that control cell growth and production. Nonetheless, it is still early to accomplish that NRF2 is an oncogene. More research is needed to explain if fundamental activation of NRF2 is enough to start cancer development. Besides, various studies have confirmed that temporary activation of NRF2 protects against chemical carcinogenesis, while NRF2 removal in mice rises tumorigenesis [126].

The activity and part of NRF2 in cancer is contentious and thus systematic researches are obligatory to find the exact role of NRF2 in pathogenesis of cancer. NRF2 activators might play a positive therapeutic role in prevention and treatment of cancer as well as molecules that inhibit NRF2 might play a beneficial role in chemotherapy of cancer.

## Data Availability

Not applicable.
